# American Dental Convention

**Published:** 1857-10

**Authors:** 


### ARTICLE III.

### *American Dental Convention.*

The Third Annual Session of the American Dental Convention was held at the Meionaon Hall, in the city of Boston, on Tuesday, Wednesday, Thursday and Friday, August 4th, 5th, 6th and 7th, 1857; commencing on Tuesday, the 4th, at 12 M.

The following is a list of the members:

*Maine.*—A. K. Gilmore, G. W. Reed and B. T. Cowin, Bath; S. B. Straw, Bangor; J. S. Chase, Hallowell; J. B. Fillebrown, Winthrop; W. Randall, Farmington; C. H. Burr, Portland; J. Mason, Saco; T. J. W. Trussell, Rockland; G. F. Waters, Waterville.

*New Hampshire.*—J. Smith and A. J. Young, Dover; J. A. Chamberlain and Joseph Austin, Manchester; J. W. Little, Concord; W. Ball, Walpole; J. W. Russell, Winchester;

Frank Fuller, E. S. Ryder and S. Baker, Portsmouth; A. Severance, Great Falls; M. M. Smith, Claremont; E. G. Cummings, Lancaster; A. M. Moore, Lebanon; D. K. Boutelle, Peterboro'; J. A. Reed, Newport; —— Locke, Nashua; S. W. Hale, Oxford; P. A. Stackpole, Dover.

*Massachusetts.*—E. G. Tucker, J. A. Cummings, J. Shepherd, E. T. Wilson, A. Ball, N. C. Keep, G. O. Stearns, H. B. Hale, S. P. Bartlett, E. Blake, J. M. Thresher, N. K. Mayo, J. Ayling, O. P. McAllaster, J. Clough, J. A. Salmon, S. Simonds, H. P. Hemmingway, J. J. Wetherbee, Boston; W. S. Phipps, Marlboro'; F. Field, Waltham; S. G. Henry, Westboro'; George O. Fairbanks, Fall River; W. H. Noyes, Gloucester; A. A. Cook, Milford; W. H. Jones, Wooster; C. E. Thompson, Groton; J. Farnum, Salem; E. Sanborn, Andover; C. Washburn, Bridgewater; Thomas Palmer and C. H. Whitney, Fitchburg; H. N. Macomber, Lynn; H. Collins, Salisbury; J. H. Batchelder and W. L. Boudoine, Salem; George N. Whitney, L. W. Puffer and E. M. Atkinson, North Bridgewater; C. F. Hein, Waterton; S. P. Miller, H. F. Bishop, O. F. Harris and J. Childs, Worcester; J. H. Kidder, Lawrence; F. Searle, M. E. Ames and C. S. Hurlburt, Springfield; D. B. Ingruls, Clinton; C. S. Hicks and C. G. Davis, New Bedford; J. McGregory, Sturbridge; J. Beales, Greenfield; J. Thompson, Sandwich; W. Cutler and D. P. Wilson, South Boston; D. S. Bartlett, Roxbury; E. E. Streeter, North Adams; Wilkes Allen and D. S. Dickerman, Taunton; J. T. Folsom, Gloucester; N. C. Fowler, Yarmouth; A. Lawrence, Lowell.

*Vermont.*—O. K. Post, Brattleboro'; E. N. Harwood, Rutland; M. Newton, Montpelier; M. Tefft, West Poultney.

*Connecticut.*—D. H. Porter, Bridgeport; L. Betts and L. Potter, New London; W. Potter, Norwich; R. G. Reynolds, Waterbury; E. E. Crowfoot, Hartford.

*Rhode Island.*—A. C. Hawes, W. N. Martin, L. N. Seeberry; R. P. Berry, Newport; John A. Robertson, Warren; H. H. Farnham, Westerley.

*New York.*—A. McIlroy, W. B. Roberts, W. Dalrymple,

John Allen, W. H. Dwinelle, B. Lord, George Clay, S. H. Clark, E. A. L. Roberts, G. H. Perine, George S. Hawes, D. J. S. Dodge, Jr., E. J. Dunning, C. S. Miles, W. H. Allen, J. M. Crowell, New York city; A. Blakesly, A. N. Priest, H. S. Nichols and L. W. Rogers, Utica; J. E. Ostrander, Oneida; U. Bachelor and J. A. Perkins, Rome; A. Blake, Sardinia; E. Ware Sylvester, Lyons; S. B. Palmer, Tully; S. W. Sutton, Green Point, L. I.; W. A. Palmer, Poughkeepsie; C. D. Hayward, Brooklyn; H. C. Grant, Lima; E. D. Fuller, Peekskill; D. S. Goldey, Oswego.

*New Jersey.*—A. W. Kingsley and S. E. Armes, Elizabeth; J. C. Robins, Jersey City; W. W. Ward and A. A. Cleveland, Newark.

*Pennsylvania.*—Elisha Townsend, T. L. Buckingham, A. M. Asay, J. R. McCurdy, C. A. Du Bouchet, C. S. Orum, Philadelphia; W. A. Chittenden, Scranton; T. J. Chandler, Rochester; John Waylan and S. Welchens, Lancaster.

*Delaware.*—H. Garrett, Wilmington.

*Maryland.*—C. A. Harris, W. H. Stinson, A. A. Blandy, Baltimore; H. H. Harvey, Hagerstown; G. S. Fouke and R. R. Booth, Westminster.

*Virginia.*—J. G. Coates, Big Lick; James Johnson, Staunton.

*South Carolina.*—S. Blanding, Columbia.

*Georgia.*—A. W. Allen.

*Missouri.*—J. Forbes, C. W. Spaulding, A. M. Leslie, St. Louis.

*Mississippi.*—H. C. Kendrick, Natchez.

*Louisiana.*—A. F. McLain, Franklin.

*Tennessee.*—T. B. Hamlin, Nashville.

*Kentucky.*—D. W. Roundlebush, Covington.

*Illinois.*—W. W. Allport, Chicago; I. B. Branch, Galena.

*Michigan.*—O. M. Carlton, Ypsilanti.

*Wisconsin.*—D. W. Perkins, Milwaukie.

*Ohio.*—James Taylor, J. Taft, Charles Bonsall, W. Storer How, Cincinnati; C. R. Taft, Mansfield; A. E. Lyman, Newton Falls.

W. D. A. and —— Brown, (no residence given.) Also, a gentleman from "Lewiston Falls," another from "Wellfleet, Mass.," and another of "Boston," whose names we cannot decypher.

Prof. Chapin A. Harris, of Baltimore, called the Convention to order.

The Secretary, Dr. Elisha Townsend, of Philadelphia, read the names of the Business Committee, by which it appeared that the chairman, and some of the other members, were absent.

Prof. Harris stated that he had received a letter from the chairman, (Dr. J. S. Clark, of New Orleans,) stating that he should not be able to be present, and the letter was read.

Dr. W. W. Allport, of Chicago, inquired if it would be expected that the committee should make a report upon the order of business.

The President stated that inasmuch as the members of the committee had probably reflected somewhat upon the business proper to be presented, he thought they would be better qualified to attend to the matter than a committee newly appointed.

The roll of the committee was then called, and it being ascertained that a majority of the committee were present, the President stated that, according to the usual course of proceeding, Dr. H. C. Kendrick, of Mississippi, would act as chairman.

Dr. Kendrick excused himself, and the subject of the appointment of a chairman was left to the committee itself.

The minutes of the last session of the Convention were then read by the Secretary.

An inquiry was made of the Secretary, if the records did not attribute the invention of an instrument, to which reference was made, for producing local anæsthesia, to Dr. Putnam, of New-York, instead of Dr. Branch, of Illinois, to whom it properly belonged.

While the Secretary was examining his minutes, the President said that he believed he could answer the question When he introduced the matter to the convention, he supposed the instrument was the invention of Dr. Putnam, and that he claimed it; but he had since ascertained that Dr. Branch was the inventor.

Dr. Wm. H. Dwinelle, of New York city, said this matter had become a part of history. Dr. Branch was the inventor, and had secured letters patent, and nobody else had any claim to the invention.

The Secretary then stated that the minutes did not give the name of Dr. Putnam as the inventor.

The President said that while the business committee were engaged, he would embrace the opportunity to state to the convention that he had received a letter from Dr. J. M. Weiber, of Paris, offering a new preparation of gold for filling teeth, which the writer believed preferable, on many accounts, to any other preparation heretofore used by the dental profession.

The letter was then read by the Secretary.

The President said it would be proper to state, that the box containing the gold and the tooth referred to in the letter, was opened at the custom house, and the tooth lost—it never reached him.

Dr. Charles Bonsall, of Cincinnati, moved the acceptance of the letter.

The President suggested its reference to a committee, with the gold accompanying it, that the latter might be fairly and thoroughly tested.

Dr. Isaiah Forbes, of St. Louis, moved that it be referred to the officers of the convention.

Dr. Townsend said this motion would meet his views much better than the appointment of a committee. The organization of the Convention was decidedly a democratic one, and he feared, if they began by appointing a committee in this case, which would seem to warrant it, they would get themselves into a kind of trouble and take upon themselves responsibilities which they ought to escape, and which they did not wish to assume, and of which undue advantage would be taken by those who were always ready to hang to the back of anything they thought strong enough to carry them. They found last year, that gentlemen came to the Convention, got what knowledge they could, and then went home and made a card of it, publishing themselves as having been to the great National Convention, and saying they had learned wonderful things, which would be put to the advantage of their patients, and put into their own pockets afterward. He did not think the Convention was established for any such purpose. They wanted to obtain and give all the information they could, but they did not want to commit themselves, by the appointment of a committee, to the condemnation or approval of anything. The mere fact of the appointment of a committee would be enough, in some cases, to afford an opportunity for parties to go home and claim credit for themselves, on the ground that the Convention had considered their inventions of sufficient importance to appoint a committee to investigate them. He wished to avoid all such difficulties.

Dr. Dwinelle said they must be careful to avoid either extreme. He did not see how it was possible for a body like that to carry out its purposes without some machinery. It would be remembered he had previously deprecated encumbering the operations of the society with very much machinery; he wished to avoid that extreme, but still, he did not see how they could get along without committees. It was assuming that there was no necessity for system.. There must be collateral matters going on at the same time with the business of the Convention, and he, therefore, favored the appointment of the committee.

Dr. Forbes said the letter was one written to the President of the Convention, in as respectful a manner as any letter could have been written, and it appeared on its face, that Dr. Weiber had an invention which he considered would be of advantage to the whole profession. The President had thought well enough of it to have it read to them, and he thought the greatest respect they could pay to the writer, as a scientific man, was to refer it to the officers of the Convention, as their most distinguished members.

Dr. Frank Fuller, of Portsmouth, N. H., suggested that a committee be appointed, to whom all matters of a similar character should be referred. He said, that in this progressive age, they would be likely to have many such things presented for their attention, and he thought the appointment of such a committee would be a great saving of time.

Dr. G. O. Stearns, of Boston, expressed the opinion that the meeting was a Convention of the Dental Profession, not a Mutual Admiration Society. He hoped every thing would be done in an open, democratic manner.

Dr. Forbes said, his only object in making the motion was to get the subject out of the Convention at that time, in order that they might go on with their business.

Dr. Branch said he would second the motion. He thought the Convention would be safe in the hands of its officers.

Dr. John Allen, of New York, said he thought they were likely to get into difficulty. If this matter was referred to the officers, it virtually appointed them a committee. This was an important question, because it would establish a precedent on which they were to act hereafter. He thought it was not the object of the Convention to press any opinion upon such matters—to build up A, B, or C, or put him down at pleasure. If he understood the object of the Convention, it was to bring the whole into the great reservoir of useful knowledge. He thought a resolution ought to pass, to the effect, that they would not endorse any improvement brought before them; but that they would examine it, each man looking upon it with reference to its merit or demerit, but not that they were to endorse or reject it as a body.

Dr. W. W. Allport, of Chicago, called to order. He said the whole discussion was out of the prescribed order of business, and he would move to lay the whole subject on the table. The motion prevailed.

Prof. James Taylor, of Cincinnati, from the committee on that subject, reported the following as the order of business for the Convention:

1st. President's address; 2d. Election of officers; 3d. Debate, "What are the best means for securing a healthy denture;" 4th. Debate, "What are the mechanical appliances necessary to secure the same;" 5th. Debate, on the "best manner of treating alveolar abscess;" 6th. Discussion on mechanical dentistry; 7th. Discussion on filling teeth. Speeches to be limited to ten minutes, unless otherwise ordered.

The committee also offer the following resolution:

*Resolved*, That all persons having mechanical means or appliances to present to the Convention, be requested not to bring them into the Convention, but deposit them in a separate room, for examination.

The report was accepted and adopted.

The President said—Gentlemen, the first business in order, according to the report of your committee, is an address from the President; but I have to throw myself upon your kind indulgence, as I have often before been compelled to do. I have no address to make. I would have prepared one, but the larger part of the time since the last meeting, my health has been such as to prevent me from writing, and it is only during the last six or eight weeks that I have been able to use my pen, and this I was prevented from doing by professional duties which had been before neglected. But I will take occasion, while up, to express the great gratification which it affords me in again having the opportunity of meeting with so large a number of my professional brethren. I am glad to see them here; I am glad that they feel so lively an interest in the advancement of dental science, as to put themselves to the trouble and subject themselves to the expense of coming from far distant and widely separated portions of the country, with a view of receiving information from their professional brethren, and interchanging views with each other upon professional subjects; and I am sure that every one will be amply compensated for this trouble and this expense in the accumulation of knowledge, for he will return from here with his hands and heart strengthened, to enter with renewed energy upon the arduous duties of his profession, and be better able to meet the wants of those who seek his aid. I have always been an advocate of dental associations. It has been said of dentists—and perhaps truly—that there was a feeling of rivalry existing among them which has heretofore prevented them from acting together, from associating with each other, from interchanging views, and this, no doubt, has retarded the progress of practical and scientific dentistry; but I believe that the time has well nigh arrived, when this feeling will be discountenanced and cease to exist; and I trust that harmony of feeling and brotherly love will characterize all the proceedings of this Convention, and that we may return to our several homes, having a more favorable opinion of each other than we have ever had before.

But I will not detain the Convention with any further desultory remarks of this kind.

[A gentleman in the hall made an inquiry with regard to the qualifications requisite to become a member of the Convention. He did not know whether he could consider himself as such or not.]

Dr. Allport said, that in sending out the circulars, he had assumed a little prerogative. He meant by "practicing dentists," to convey to those who received the circular, the idea that any one could become a member of the Convention who wished. He did not mean that every one could vote without complying with the requirements of the Constitution. It seemed to him proper that every member should sign the Constitution, and pay his proportion of the expenses.

Prof. Taylor, of Cincinnati, said, that at the last meeting this subject was thoroughly discussed, and he thought they did their best to make every dentist in the United States, Europe, Africa and Asia, understand that he was a member as soon as he came here; and that fact they published to the world. So far as the signing of the Constitution was concerned, he thought that was done simply to get the names of those present, and no man was compelled to pay one dime towards the expenses. They took it for granted, and he hope they always would, that that body of dentists was ready and willing to meet all the expenses that might be incurred. (Applause.)

Dr. Townsend said, he perceived they wanted an open Convention—more open than it was. He would, therefore, move that they do away with the Constitution altogether, and invite every dentist, every where, to become a member.

Dr. Dwinelle seconded the motion, and hoped that it would include not only practicing dentists, but all who had ever been practicing dentists. If their excellent President should leave the profession to-morrow, he could not be a member of the Convention. Though a *practical*, he could not be considered a *practicing* dentist.

Dr. Townsend said, he should like to see gentlemen connected with other scientific professions in the Convention. He had learned a great deal in reference to his own profession, from men who knew nothing at all about it. (Applause.) They had given him the results of their investigations, and he made an application of them which they did not dream of.

Dr. Spaulding, of St. Louis, said the suggestion met his hearty approval. He was glad that it had been offered, for it seemed to him difficult to fix the qualifications that should render a man a member of the Convention. He thought they should place it on the broad ground that any man who professed to be a dentist should be entitled to membership.

Dr. J. A. Perkins, of Rome, N. Y., stated that he had been a practicing dentist, but was not now, and inquired if he could vote. The President replied that he could.

Dr. Townsend's motion was adopted, and Dr. T. then said, that this was what he aimed at two years ago, but to speak the whole truth about it, he was afraid to undertake to start the movement, without something that looked like a piece of red tape to tie up the members. He was afraid he could not get men to come to that platform without it; but he wrote the Constitution, so that it was bind and loose, bind and loose all the way through. It seemed, however, that it bound enough to make some cavil, and, therefore, he voted to throw it away.

The Convention then proceeded to the second item in the order of business, namely, "the election of officers."

Dr. F. H. Clarke, of New York city, moved that the Convention proceed to ballot for President, the three gentlemen receiving the highest number of votes on the first ballot, to be considered the candidates, the lowest on the second ballot to be dropped, leaving only two names for the final ballot. The motion was adopted.

Drs. Allport and Roberts, were appointed tellers.

After the first ballot, Prof. Taylor, of Cincinnati, Drs. Allport, of Chicago, Forbes, of St. Louis, and Townsend, of Philadelphia, were declared the candidates, the two former and the two latter having received tie votes.

Dr. Townsend stated that he should decline being considered as a candidate, as he should not be able to attend the next Convention, as he expected to be in Europe at that time.

On the second ballot, Prof. Taylor, had 52 votes; Dr. Allport, 26; Dr. Townsend, 15; Dr. Forbes, 4.

On motion of Dr. Dwinelle, the election of Prof. JAMES TAYLOR, of Cincinnati, as President of the Convention, was made unanimous.

Dr. Bonsall, of Cincinnati, suggested that as they had taken a western man for President, they take an eastern man for Vice-president.

Dr. Fuller, of Portsmouth, N. H., said it seemed to him highly proper and courteous that the two extremes of the country should be represented in the two highest offices of the Convention, and he would, therefore, nominate as a candidate for Vice-president, Dr. Straw, of Bangor. He was one of the oldest members present.

On the first ballot, Dr. E. G. Tucker, of Boston, received 42 votes; Dr. John Allen, of New York city, 29; and Dr. Straw, of Bangor, 18, and the two former gentlemen were declared the candidates.

While the committee were collecting the votes on the second ballot, Dr. Rogers, of Utica, N. Y., moved that when the Convention adjourn, it be to meet at four o'clock in the afternoon. Adopted.

On the final vote for Vice-president, Dr. Tucker received 52 votes, and Dr. Allen, 34, and Dr. Tucker was declared elected.

On motion of Dr. Dwinelle, the election was made unanimous.

*Afternoon Session.*

The Convention was called to order at 4 o'clock, by the President.

The election of officers was proceeded with, Dr. J. R. McCurdy, being appointed on the committee, to collect and count votes, in place of Dr. Allport, resigned.

On the third ballot for Recording Secretary and Treasurer, Dr. L. W. Rogers, of Utica, N. Y., received 38 votes; Dr. Spaulding, of St. Louis, 31; and Dr. Rogers was declared elected, and the election made unanimous.

The Convention then proceeded to ballot for Corresponding Secretary, and on the third ballot, Dr. Isaiah Forbes, of St. Louis, was declared elected, he having received 47 votes, to 33 for Dr. Johnson, of Virginia.

Drs. Allport and Perine, were appointed by the President to conduct Prof. Taylor, President elect, to the chair.

Prof. Harris, on leaving the chair, said—I have to convey to the Convention my hearty thanks for their indulgence; and I am gratified that it has made selection, for its presiding officer, of a gentleman with whom I have been acquainted for many years—from the very commencement of his professional career. Indeed, we started out from the same school; we had the same teacher; and although we have been somewhat widely separated from each other in the exercise of our professional duties, I believe we have always worked harmoniously together. (Applause.)

Dr. Allport then said, that in behalf of the President, and as a western man, it gave him exceeding pleasure to introduce to the Convention the President, Prof. Taylor, of Cincinnati.

The President, on taking the chair, spoke as follows:

"Allow me, gentlemen, first to reciprocate the very kind feeling expressed by the gentleman who has just left the chair. He has alluded to the past—to a period of life when we were young in our profession, when, I was about to say, we scarcely had a profession. He has brought to mind those feelings and memories, which, in addition to the kindness you have this day manifested towards me, have indeed done more to throw me completely beyond the power of speech, than anything which he could have done. (Applause.) There are feelings striving for utterance, which language fails to embody in words; there are emotions gushing up from the deep fountains of my heart, which rush out with such force that I cannot form them into proper words for this occasion.

"We have met, gentlemen, for one very important purpose; may I not say for two very important purposes? First, we have met for the advancement of dental science. We have met to investigate every truth on which that science is founded. We have met to see if those rocks of truth on which the foundation of our temple is now being erected are firmly cemented together. We have met, gentlemen, to compare notes; we have met to exchange ideas with each other, and to refute or confirm opinions previously entertained. We have also met, may I not trust, to impart to those assembled with us here useful knowledge; and may I not express the hope that every member of this convention will be ready to impart all the information in his possession, that we may all be benefited thereby.

"We have met also for one other important purpose. We have met to renew old acquaintances; to draw more closely those bonds which bind us to each other, and the profession we esteem. We have met to interchange not only our views but our sympathies; and we have met also to gaze for the first time upon the faces of those whose works and writings have been speaking to us for years, but who now, for the first time, are brought within our cordial embrace. Who would not be here? Who would not lay aside the labor and toil of his profession to come to this eastern temple to usher in, as it were, a new science—the last mark of man's progress in dental and scientific knowledge? Who would not participate in such a noble work? Gentlemen, by your kind action in selecting me as your presiding officer, you have conferred upon me an honor that I know I cannot merit, and I feel utterly inadequate at this time to the task imposed upon me; and did I not know that you are all animated by a thirst for knowledge which will lead you to set aside the formalities of parliamentary proceedings, I should shrink from the chair I now assume. I shall, therefore, ask your kind indulgence and hearty co-operation, in order that our deliberations may be such, that this new science, if I may so call it, may continue to advance from one step to another until it ranks with that ancient and time-honored profession which we all revere and respect." (Applause.)

Dr. Perine then offered the following resolution, which was adopted, the Convention unanimously rising:

*Resolved*, That the thanks of this Convention be tendered to Prof. Harris for the very impartial manner in which he has presided over its deliberations.

A vote of thanks was also passed, on motion of Dr. Allen, of New York, to the other retiring officers, for the faithful manner in which they had discharged their duties.

Prof. Harris then moved the appointment of a committee of five, to examine the gold sent to him by Dr. Weiber, of Paris, the letter accompanying which was read in the morning. He said it was possible the gold might possess valuable qualities, if so, the profession should know it, and make a suitable acknowledgment of its receipt.

Dr. Spaulding suggested that the committee be made a standing committee, to whom should be referred all similar matters during the session.

Prof. Harris said he should have no objection.

Dr. Townsend expressed the opinion that this should be a special committee. It had come to them in a little different manner from anything else they were likely to have, and he hoped it would be referred to a special committee.

The committee was then ordered to consist of five, which the chair appointed, as follows: Drs. Dwinelle, of New York; Allport, of Chicago; Townsend, of Philadelphia; Taft, of Cincinnati; Tucker, of Boston.

On motion of Dr. Lord, of New York, the committee were requested to report during the session of the Convention.

Dr. Fuller, of Portsmouth, N. H., offered the following resolution:

*Resolved*, That all members of the medical profession, chemists and metallurgists be, and the same hereby are, cordially invited to attend the meetings, and to take part, if they please, in the deliberations of this Convention.

This resolution called forth some little debate, and was finally amended by striking out all after the word "meetings," in which shape it was adopted.

Dr. Bonsall, of Cincinnati, moved that the Convention adjourn to meet to-morrow morning at 10 o'clock.

A motion to substitute 9 for 10 o'clock was adopted, and the Convention adjourned.

*Second Day.—Forenoon Session.*

The Convention was called to order, on Wednesday morning, at twenty minutes past 9 o'clock.

A brief discussion took place on the subject of finances, which terminated by the appointment of Drs. Allport, of Chicago, and Miller, of Worcester, a committee to take the names of members and collect funds.

The Convention then took up the first question for debate: "*What are the best means for securing a healthy denture?*"

Some hesitation being manifested in opening the debate, the President called on Prof. Harris to present his views to the Convention; but that gentleman declined, stating, however, that at a later stage of the debate, he might have something to say.

Dr. Baker, of New Hampshire, read a communication published by him in the Portsmouth (N. H.) *Journal*, on the effects of saleratus, cream of tartar, and carbonate of soda, on the teeth. He regarded the use of alkalies in the making of bread as highly injurious, and looked upon them as a principal cause, (though not as the only cause,) of diseased teeth. In answer to an inquiry, Dr. Baker said he thought these substances affected the enamel of the teeth, but not so much as the softer bone.

Dr. Spaulding, of St. Louis, confessed that the assertion that alkalies were injurious to the teeth was a new idea to him; and, in connection with this matter the question arose—Is saleratus a pure alkali? It was well known that saleratus was carbonate of potash, that carbonic acid gas enters largely into its composition. The question was, then, is its action purely that of an alkali? In laying down principles, they must be sure they were strictly correct.

Dr. Kendrick, of Mississippi, inquired through what medium the alkali affected the teeth.

Dr. Baker. Through the medium of the food with which it was mixed.

Dr. Kendrick. Why don't it affect all teeth alike?

Dr. Baker. Some eat more than others.

Dr. Kendrick said that was true. He regarded this subject as a most important one, because it aimed at preventing the trouble they were all united in trying to correct. They were often pained by having children brought to them of eight or ten years old, to have permanent molars taken out, whose parents were perfectly thunderstruck when told that they were the permanent teeth; they had supposed they were the temporary organs, and had been careless of them. He thought the cause of decay was the accumulation of extraneous matter about the teeth, whether of animal or vegetable food; and if he were to answer in a single word the question, "What are the best means of securing a healthy denture," he would say—CLEANLINESS. (Applause.)

Dr. Perine said this was a very important subject, and he was glad it had been brought before the Convention. He had come there, with other younger members of the profession, to learn, and he would be glad to hear from Prof. Harris on this subject.

Prof. Harris said, if he had consulted his own feelings, he would have preferred to remain a listener; but, at the request of several of his professional brethren, he rose to say a few words upon the subject. He could very well conceive that bread, having in its composition saleratus and cream of tartar, if it injured the general health, might indirectly affect the teeth prejudicially; but that they had any direct action upon them, he could not for a moment believe; for the reason that the alkaline properties of the saleratus were neutralized by the cream of tartar. That caries of the teeth is the result of the direct action of alkalies, is refuted by the fact that the animal frame-work remains, and even with exalted sensibility, long after the destruction of the earthy salts of the affected parts. But so far as particular kinds of diet and modes of living were capable of affecting the general health, so far the liability of the teeth to decay might be increased. He was of opinion that if every individual came up to a perfect standard of health, caries of the teeth would very rarely, if ever, occur, for the reason that they would be capable of resisting the action of those chemical agents, to the presence of which caries of the teeth was now universally admitted to be attributable, more especially, if the ordinary means of cleanliness were resorted to. He agreed with his friend, Dr. Kendrick, that cleanliness was the all-important thing for the preservation of the teeth ; but some teeth were so susceptible to the action of chemical agents, so imperfect in their physical condition, that they were very easily assailed. For instance, in teeth of a very soft texture, deep indentations were found in the grinding surfaces, sometimes absolute imperfections of the enamel, through which the chemical agents of the mouth, whether existing in the fluids of this cavity, or were the result of some change which takes place in the remains of alimentary substances lodged in these indentations or between the teeth, are introduced. Teeth of this description decay easily and rapidly. Again, they oftentimes meet with instances of caries, or erosion, if they pleased to call it such, immediately after the eruption of the teeth from the gums, showing that it was to the action of the fluid contained in the sacks of the teeth, and that, too, after they had become completely enameled, that decomposition was attributable. Several examples of this kind which had come under his observation, occurred to him now. In one instance the destructive ravages of which ran through a whole family, and seemed to be dependent upon some peculiar constitutional idiosyncrasy. In almost every member of that family, the teeth, the molars particularly, were found to be more or less eroded immediately on their eruption from the gums, and before they were brought in contact with any agents from without. The fluids of these sacks were undoubtedly acidulated, and this acidulation depended upon some peculiar cachectic habit of body of the various members of the family to which he referred. He could call up a hundred examples of this kind, but one would suffice.

Again : they found a somewhat analogous process occurring in the destruction of the roots of the temporary teeth. This had usually been attributed to the action of the absorbents, but experiments had shown that the carneous substance which is brought in contact with them, before the destructive process commences; exhales an acidulated fluid, which has a stronger affinity for the lime of the teeth than the phosphoric with which it is combined; and it was usually regarded as unphilosophical to attribute to two causes an effect when one alone was sufficient for its explanation; and in this instance he supposed that the acid exhaled or thrown out from this carneous body, coming in contact with the roots of the temporary teeth, was the chief agent in breaking down the calcareous material; then the absorbents might come forward, take up the residuum, and carry it back into the general circulating system. He could not conceive that vessels so minute and delicate as the absorbents, were capable of rasping down a substance as hard and resisting as the roots of teeth.

Dr. Clark, of New York, requested Prof. Harris to touch upon the fact, that in many mouths, the caries commences at the exact edge of the gum. It seemed to him that the cause might be found in the secretions of that particular part of the mouth.

Prof. Harris said that was undoubtedly the explanation. The suggestion just made went most strongly to establish the correctness of the views he had advanced; for where the mucous secretions of the mouth were first brought in contact with the teeth, and especially where they were longest retained, was the spot where they most frequently discovered the first evidence of their destructive ravages. This is especially the case upon soft teeth—teeth easily acted upon by chemical agents. He was aware that a variety of theories had been propounded with regard to the cause of caries of the teeth, but in his opinion, it was the result of the chemical decomposition of the earthy salts of the affected part, sometimes accompanied, but more frequently followed by the disorganization of the animal frame-work of the afflicted portion of the tooth.

The question was often asked, "why are seafaring men, and persons residing near the sea-shore, more subject to caries of the teeth than persons residing in the interior of the country?" He had often remarked the fact, and he had often heard officers of the navy say, that since they had been almost constantly on the water, their teeth had decayed much more rapidly than before. He had been unable to offer any explanation, but two or three years ago, while at Cape May, he observed, after he had been there two or three days, that the locks of his trunks were very singularly affected, having turned green. It seemed to him that this must be attributable to some atmospheric influence—to some acid, perhaps; but where did the acid come from? It is well known that the ocean contains salt, a muriate or hydrochlorate of soda. Was it not possible that the evaporation going on continually on the surface of the ocean might set free, to some extent, hydrochloric acid, which, by diffusing itself abroad in the atmosphere, would be brought in contact with the teeth at each inspiration; and might not this act prejudicially upon these organs? and was it not to the presence of this acid that the change to which he had referred was attributable? This seemed to him a very plausible and rational explanation.

Dr. Dillingham, of Boston, said he had lived for some years in the vicinity of salt water, and he had frequently noticed the decayed condition of the teeth of seafaring men. On one occasion, while practicing at Edgartown, a gentleman called on him to have his teeth examined before going on a whaling voyage. He had never seen a more perfect set of teeth than that man possessed. He requested him to call and see him on his return, and some three years afterwards he did so. Five of his teeth had entirely decayed, leaving nothing but the roots, and there were some six or seven that needed to be plugged. He had endeavored to ascertain the cause of this, but had as yet arrived at no definite conclusion. He thought it might be referable to the use of salt provisions, and the vinegar and limes which were used so freely by seamen to prevent scurvy.

Dr. Baker said there was probably no man in the country who had received greater benefit from the writings of Prof. Harris, or who entertained for him a more profound respect, than himself; but on the question of the influence of alkalies on the teeth, he could not agree with him. He thought the absence of cleanliness in the case of sailors was a great cause of the decay of their teeth. It had been said that in the preparation of bread, the acids and alkalies neutralized each other; that was true, but they destroyed the goodness of the wheat, and might, in this way, act upon the teeth.

Dr. Branch, of Illinois, said that he thought there were few who would maintain that alkalies, moderately applied to the teeth, would be productive of injury. He should look further than that for the cause—in the constitutional effect. If there was an excess of alkali, as there often was, in the bread we use, that being taken into the stomach, excited that organ to throw off an extra quantity of acid. If they attempt to overpower nature in any direction, she would resist and perhaps overact in an opposite direction. Thus it was that by taking alkalies into the stomach, we excite the production of acid in the system, and the vessels are excited to an extent that becomes injurious, the acid thus produced acting prejudicially upon the teeth. Thus the alkali taken into the stomach affects the teeth, not directly, but indirectly. The great secret of a healthy denture was a healthy constitution. Give him a constitution able to appropriate, as a healthy constitution is able, all substances taken into the stomach to the building up of the system, and he would show them a healthy denture. It was sometimes asked what food was best calculated to produce a healthy denture, and that was very proper with respect to a diseased subject; but he maintained that it was little matter what food a person ate, so that it contained any proper nutriment for the body, if he had a healthy constitution, to assimilate the food to the building up of the system. Their attention, as dentists, in this matter, should be directed to those things which were calculated to give their patients, whether young or old, a healthy constitution. He should answer the question, "How shall we produce a healthy denture?" in one word—"produce a healthy constitution."

Dr. Reed, of Newport, N. H., said he had been instructed and edified by the remarks of the speakers who had preceded him. It had been correctly stated, in his opinion, that a person having a healthy constitution would have a healthy denture; but their patients were thrown upon their hands with an unhealthy constitution, and he wished to know the best means at their command, for securing a healthy denture to those persons, notwithstanding the unhealthy constitution with which they found them. They had young patients brought to them daily, even infants in their mother's arms, to know what should be done to secure for them good teeth. He believed that acid was the great enemy they had to combat, and, therefore, he had uniformly directed the use of a mild alkali, to neutralize not only the acids of the fluids of the mouth, but the acids which are produced by the fermentation of the foreign substances which may be lodged between the teeth, in cavities, and beneath the gum, especially upon the buccal muscles, where there was no action of the tongue to remove them. He recommended his patients to use a little nice soap, and cleanse the mouth thoroughly.

Dr. Clark, of New York, said this was a subject of extreme interest to him, and he wished to say a few words, that might call forth remarks from others more capable of elucidating the subject than himself. He felt convinced that the cause of decay upon the surface of the teeth, on the line of the gum, and particularly on the outside of the gum, could not possibly be owing to any secretions there, for the reason that he had cases in his own family, where these places had been cleansed often, and it had been found almost impossible to prevent decay there; and this, too, when all the other teeth even where they were crowded, and it was impossible to clean them, had manifested no signs of decay. He was of opinion that the cause of decay there was different from that which produced decay in the grinding surfaces of the molar teeth, and he hoped this discussion would be protracted, that they might have the views of other members of the profession upon the subject. The case seemed to him analogous to the decay of many vegetable substances, the decay of posts in the ground, and the decay of vessels, where there was an alternate exposure to atmospheric influences and to moisture. It seemed to him that there must be an acid generated at the edge of the gum, peculiar to the place, which, at the moment of its production, acts directly upon the teeth. There were many cases that came to the knowledge of every dentist, where they found that those surfaces were polished and polished, until they were polished through, but still the same action was manifest upon the enamel, and he believed it was almost exclusively confined to the enamel.

Dr. Allen, of New York, said the great object of the Convention was to arrive at facts, and see if they could not do more good in their profession. This was what their patrons and the community demanded of them ; and he believed they had at length struck at the root, the very starting point of improvement. As to the cause of decay, they had been told that if they had a healthy constitution, they would have sound and healthy teeth. He subscribed to that ; but here was the question—"How should they secure that healthy constitution ?"

Dr. Branch, (interrupting,) said that in the remarks he had made, he meant to be understood, that in order to produce a healthy constitution, or to make a diseased constitution healthy, they should study constantly to keep up the balance of the system, by exciting alternately the production of acid and alkali. It was no use to undertake to subdue acids in the system by putting alkalies into the mouth.

Dr. Allen said that was the point he was attempting to reach. It was their duty, as he conceived, to pay most especial attention to those causes which would produce the breaking down of a good constitution. They had been told that it mattered little what they ate, provided they could maintain this balance ; and the proviso was well thrown in. In the West, where there were a great many distilleries, cattle and hogs were fed on distillery "slops" to a great extent. If a cow, that had been raised on grass, was fed ten years on "slops," her teeth would become so affected that she could not maintain life by eating grass. If the animal economy was thus affected in the case of the cow by her food, why not look at their own systems and see whether they were not affected in the same way—whether the same law does not govern us as the cow or the hog. He took the ground that caries of the teeth was the effect of chemical action, produced by the superabundance of acids or alkalies, the one affecting the limy portions, the other the gelatine portions of the teeth. The point was to discover what kinds of food will produce the most healthy condition of the system. He thought that if they were to live on a more simple diet, if they had less to do with acids and alkalies, they might look for better teeth. He thought there was a law in the animal economy that would teach them a good lesson.

Dr. Sylvester, of New York State, referred to a case cited by Dr. Baker in the article read by him, of several students, who had been very seriously affected by the bread they ate, which was mixed with saleratus. He said he knew something in relation to that matter, and that it was not the saleratus alone which had produced the effects spoken of. The boarding house where the students lived was a cheap one, where the object was to procure the greatest amount of nutriment for the least amount of money. A great portion of their food consisted of gingerbread, sweetened with cheap molasses. It was the sweet and the saleratus, occasioning a disordered stomach, that produced the disease, not the saleratus alone.

He was glad to hear the question brought up with reference to the decay of the teeth of seamen, but he thought they must look for the solution of the cause in another direction from that mentioned by Prof. Harris. He thought he could make this evident by one simple statement of fact. Some years since, having his attention turned to the effect of diet upon the teeth, he determined to make an examination into the condition of the teeth of the aborigines of our country, and accordingly visited the sea coast near the city of Darien, Ga., and spent a day in exhuming the remains which had been there thousands of years, for all he knew, and collected a bushel of teeth, among which he found only one case of disease, which resulted not from caries, but from the wearing down of the tooth until it reached the pulp cavity. Those aborigines lived right on the sea coast; the mounds from which the teeth were taken were not more than half a mile from the sea; so that he thought, if it should prove a fact, that people living on or near the salt water, are more subject to caries of the teeth, than people living inland, they must look for the cause in another direction than the influence of salt air upon them. It seemed to him that they might go back even further than the preceding speakers had gone in seeking to account for this fact. It was important for them to tell those who were becoming mothers, that even before birth, the teeth in embryo exist in the infant, and that causes which influence their formation at that time may have their effect years afterwards. How was it with our ladies? With compressed systems we have a compressed alveolar ridge. If they would effect a change in this matter, they must go back to the primary cause, and ask the women of the country to dress and live according to nature, and then, when their children died, their teeth would be like those of the aborigines of the country, and their profession useless. The manner of living affected the teeth gradually. He had noticed that persons coming to this country from England, with perfectly sound teeth, after living here a few years, found their teeth begin to decay. That it was not the climate, was proved by the fact that the aborigines had sound teeth. It was their mode of living—their hot food and saleratus.

Dr. Dwinelle said he thought he could answer the question now under consideration, in one word—*cleanliness*—positive and absolute CLEANLINESS; studying the necessities of the constitution, and contributing to them in such a manner, that a healthy condition of the secretions of the system will be secured. It was well known that their constitutions were continually changing more or less, from one extreme to the other. They had only to study these changes, and consider that they were, in fact, chemical laboratories, containing every element within themselves that was embraced in the universe, and they had only to use their common sense, and study to regulate things correctly. It seemed to him that nothing was more true, than that the decay of the teeth came purely from chemical action, and was owing to an excess of acid or alkali in the system, the acid acting upon the lime, the alkali upon the gelatine. They had only to go into a chemical analysis of their teeth and bones, to ascertain where the trouble lay. He presumed there was not an individual present, who had not prescribed alkalies, under some modification, to his female patients. He knew that the teeth of females, during one period of gestation, decay more rapidly than at any other period of their lives; they must correct this. He did not think that saleratus decayed teeth. They were continually taking substances into their mouths, which, if used exclusively, would destroy the teeth. Dr. Westcott had been over this entire field, and proved to a demonstration, just what effect each individual substance had upon the dentition. It seemed to him, that cleanliness, and a careful attention to the idiosyncrasies of the individual constitution—because what was applicable to one individual was not applicable to another—were the great requisites for securing a healthy denture.

Before sitting down, Dr. D. said, he desired to refer to the remarks made by his friend, Dr. Sylvester. His intimation was, that the aborigines were not afflicted with decayed teeth. It was the general and popular opinion that we were a degenerate race. He begged leave to differ; we were a changing race, but he believed not a degenerate race. He believed we had as good specimens of men, physically, in these latter days, as ever were known in the days of Moses or Noah. His observation differed materially from that of Dr. Sylvester. He happened to be born—accidentally and without consultation—(laughter) in the upper part of New York State, where many Indians were buried. He had not dug up Indians by the hundred, nor gathered teeth by the bushel, but he had desecrated fifty graves, perhaps, and in his perambulations, he had found a great many decayed teeth. He did not know but his predecessors then might have used an excess of saleratus, (laughter) but he only spoke of the fact. He had also in his possession the tooth of an Egyptian mummy, which was very badly decayed, and showed plainly that it troubled its owner, who might have been cotemporary with Noah or Moses himself, with the toothache. We had, too, he believed, an unquestionable specimen of Egyptian dentistry—the tooth of an Egyptian had been found filled. He did not believe, therefore, that we were a degenerate race. In fact, he was not prepared to say that the toothache was not among Job's afflictions.

Dr. Palmer, of Poughkeepsie, was not prepared to deny that the use of saleratus was one of the causes of the decay of teeth, but he believed the causes were multitudinous. He could not altogether agree with the preceding speaker, in the opinion that the teeth of the present generation were as good as those of the preceding. He thought the matter of diet, was one that called for their consideration. The question under discussion opened a wide field, and he thought a powerful influence might be exerted upon the community by the discussion there, and much good be the result.

Dr. Priest, of Utica, thought dentists were disposed to ride hobbies. There was no single cause of the decay of teeth. He was often asked, "Is it candy?—is it pickles?—is it sugar?" He had only time to answer, there is no special cause, but a general cause. This cause reached as far back as the influence of the mother. Our children come to us with decayed teeth almost from their birth. It seemed to him, that they should go back to first principles in this matter, and not attempt to refer it to eating any particular kind of food, but strive to get at the principle that underlies the constitution of the patient.

Dr. Frank Fuller, of Portsmouth, N. H., thought it was about time that the old humbug of the injurious effects of soda and saleratus was exploded. That theory was every now and then broached, notwithstanding the assertion of Dr. Harris, in his "Principles and Practice," and in dental publications, that the destruction of the teeth was certainly caused by acids, and not by alkalies, which could not possibly affect phosphate of lime, upon which their strength, although not their vitality depends. Did mild alkalies, such as soda, or saleratus, placed in contact with the teeth, injure them? He would admit, that if they were macerated in a strong solution, it would soften the enamel; but who did this? Did any body keep saleratus or cream of tartar, in this form constantly, or for any number of hours, in contact with the teeth? Certainly not. They were used simply to make bread, and other articles composed of the various serials. Cream of tartar was mixed with saleratus, carbonic acid gas was generated, and this was stirred up in the bread. In the process of baking, the carbonic acid gas was thrown off, leaving simply a small proportion of Rochelle salt. Did that injure the alimentary passages, or the digestion, in the small proportion in which it was used—the 30th part of a grain, perhaps? By no means. Rochelle salt, as they well knew, was often recommended as a slight laxative, and in his opinion, it would be one of the best ingredients to mix with bread. The tendency of bread was to constipation. If a dog lived on it thirty days he would die; there was no action of the bowels. He held that saleratus and soda did no injury to the teeth. He was very glad, when one of the preceding speakers stated, that he was in attendance on one of the students, referred to in Dr. Baker's article, who partook of the "specific gravity pudding," because it assured them, that the last stone on which that article was founded, had fallen. It was plain, that it was not simply the saleratus that occasioned the sickness and death, but the cheap living, which degenerated the whole system—their cheap molasses in the gingerbread, which generated acetic acid by acetic fermentation. It was his opinion, and one which he believed would be sustained by ninety-nine out of a hundred of the audience, that the great cause of the destruction of the dental organs was acid, mineral or vegetable. Cleanliness was, indeed, a thing that they should always recommend; but this would not always save the teeth. Some teeth were so imperfect in construction, had so little earthy material, that they were acted upon much more easily than others. He had often seen young ladies and gentlemen, from ten to fifteen years old, with their superior incisors almost destroyed. Cleanliness would not remedy this evil. It would do much, but the theory should be generally understood, and he hoped that the Convention would set that matter at rest—that acids act upon phosphate of lime, but that alkalies do not, and that if we keep the teeth free from acids, and from extraneous substances, by a careful attention to cleanliness, we do all we can, in the first instance, towards securing sound and healthy teeth.

In conclusion, Dr. Fuller said, he desired to call upon his friend, Dr. Severance, of Great Falls, to explain to the Convention a new theory he had adopted, which he (Dr. F.) denominated "dental gymnastics."

Dr. Severance said his idea was simply, that the teeth, like the other organs, required exercise for their development and strength. This should be secured by a proper attention to the food, care being taken to provide such as would, in the process of mastication, afford sufficient exercise for these organs.

Dr. Townsend had been very glad to hear the discussion, but he thought they did not go far enough back. Theories were very important, for there could be no correct practice without an approximation, at least, to a correct theory. He thought they could not reach the root of the difficulty unless they could go as far back as the king of Prussia did, who passed an edict that such and such persons in his realm should not marry nor cohabit. Two persons whose constitutions could not assimilate, had no right ever to come together. He believed that feebleness of constitution produced feebleness of teeth. It was sometimes said that, in a few years, dentists would not be needed, as people were taking care of their teeth. The youngest member of the profession need have no fear on that ground. As long as persons come together, merely from a fancy for the person, there would be work enough for dentists. How were they in any manner to remedy the evil? They all knew, that in the flour they used, the hull was taken out, and only the starch—he might almost call it—left. It was the outer crust of the wheat or corn that was intended to produce bone, and that had been taken off. The consequence was, that they had less bone, and the bones were not of solid texture. He had often been consulted by ladies, with regard to the decayed teeth of their children, two or three years old, and he had sometimes said to them—what might almost seem impudent, but he did not mean it so, and they understood it—that they had not made their children well. He believed, from an experience of twentyfive years, that the main cause of decay was to be found in the formation of the teeth. They all knew that it was just at the folds of the enamel that the decay commenced in molar teeth, and that it was in the interstices between the teeth, at the margin of the gum, and perhaps a little beneath, that caries commence in the incisors, cuspids, and bicuspids. Wherever there was a bad formation, and a want of cleanliness, they had this result—the chemical decomposition from the acidified food, and from the acids of the fluids of the mouth, became concentrated at these points, where the extraneous substances could not be removed. Here, it seemed to him, was the true cause of caries. Where they found a perfectly developed body, they would find, in a majority of cases, a perfectly formed set of teeth. This perfectness of formation, as it gave to the body grace, strength, agility, power, gave, in the same way, tone, strength, smoothness and capability of endurance to the teeth. The first difficulty he had mentioned, could not be reached; they could not prevent men and women from marrying and having children whose constitutions were not adapted to each other, and they must do the best they could to arrest the evil after it was formed. He wished the profession to discover some method of prevention of the injury by acid fermentation, even in the worst formed mouth. If they could so arrange the diet of their patients, that it should contain just the proper amount of alkalies, and tend to produce a perfect dentition, they would perform one of the greatest services to the community that any set of educated and scientific men could possibly do. He hoped to live to see the day when this should be accomplished. He trusted that many of the young men who graduated at our colleges, would turn their attention to what he might call chemical dentistry; for many of them were apt to take hold of manipulation, and become indifferent to everything else. He did not want them to be satisfied with routine work, when they ought to go forward.

Dr. Stackpole, of Dover, N. H., said he thought they would all agree that it was as impossible to lay down any specific, special rules in dentistry, as in the treatment of disease. Good, common sense was the great thing to be exercised every where; they must take things as they were. He knew they could go very far back, and see very remote causes, but it was not always that they could give advice. If a lady, in the bloom of youth, came into his office, and he saw that her teeth were imperfect, it would be a very delicate matter to say to her that she ought not to be married, or to tell her lover that he would commit a great wrong by making her his wife, because, if they had offspring, they would have bad teeth. They had, therefore, to take things as they were, and so far as they could give advice on general principles, so far they would do good. His advice would be of a general character; take care of the general health, live in accordance with the laws of nature, (so far as they can be ascertained,) and seek to develop the system in all its parts, then the teeth will be developed perfectly. He had no theory to establish or overthrow about acids or alkalies, but if they saw any error in the system, if they saw anything in the laboratory indicating too much acid, or too much alkali, let them, as far as possible, correct it, and they would improve the general health, and that would have a tendency to promote a healthy condition of the teeth.

Dr. Waters, of Waterville, Maine, expressed his concurrence in the general views presented by Dr. Townsend, but not altogether. He had told them that bread was made out of a substance from which the bony material had been thrown aside. There were two substances found in man in greater proportion than any other—he referred to phosphoric acid and lime. Bone material must have phosphoric acid in order to be bone; the germ of the wheat, he apprehended, contained that phosphoric acid more than the hull. He did not know that this was so, but he had theorised himself up to this point. He conceived that we existed before there was any action of the stomach, and before the stomach was formed, and that the nervous organization was the first that had any partial completion, and that it must have a completion in the germ before any other part of the organism could be elaborated. That was his theory, and he wished those persons who had a deep insight into theorising, would take this matter up. Could nervous matter be generated without the presence of phosphoric acid? Did not that acid, in its combination with certain other acids, always manifest itself in nervous action? In order to get at this, it would be necessary to understand, what was generally conceded by physicians, that when a person undergoes extreme nervous agitation, from whatever cause, phosphate of magnesia is the result; and if this is not carried off through the urine, the patient suffers from neuralgia.

At this point, Dr. Waters gave way for a report from the Business Committee, who recommended that the discussion on the subject under consideration, terminate at half-past twelve o'clock, (in five minutes,) and that the remainder of the forenoon be devoted to the subject of filling teeth, and the afternoon session to a discussion on the best method of treating alveolar abscess.

Without taking the question on the report, the Convention voted to lay the question under debate on the table, with the understanding that it should be resumed at a future time, and took up the subject of filling teeth.

Dr. J. A. Cummings, of Boston, here announced that the dentists of Boston had made arrangements to invite their friends from abroad to an excursion down the harbor, and wished them to hold themselves in readiness at 2 o'clock on Thursday afternoon. (Applause.)

### *Discussion on the best mode of Filling Teeth.*

Dr. Taft, of Cincinnati, suggested, that in the discussion of the subject now before the meeting, each person should select such a case as they might choose, and describe their method of operating throughout. This method would bring out many things that in a more general discussion would be overlooked.

The suggestion of Dr. Taft was agreed to.

Dr. A. Blakesley, of Utica, N. Y., commenced the discussion. He selected as his case for illustration, the approximal cavity of an incisor. He commenced by examining the cavity carefully, and then removed, as neatly and thoroughly as possible, the decayed portions. His next object was to form the cavity; and in doing this, he used instruments peculiar, perhaps, to himself, principally the old-fashioned English broach, in little handles, with the ends made a cutting point, in the form of a chisel. After he had formed an angle, say of eight or ten degrees, and a little dowel-shaped point on the upper and lower surfaces, and on the side of the base, he formed a little groove, from one point to the other. Then carefully running along under the front surface of the enamel, he formed a little depression, in a rounding manner, until he approached the lower or cutting edge of the tooth. When they reached that point they will generally find that they could make another little dowel-shaped point there. Then he directed his attention to the labial surface of the tooth. In many cases, the enamel was broken away, and the tooth so far decayed, that he could not form a groove or point anywhere, and then he had to depend upon the base he made where he commenced, and in the end of the tooth where he terminated. Something might be gained by a groove from point to point in the labial surface, from end to end.

Having described his method of preparing the tooth for filling, somewhat more at length, Dr. B. then gave a very interesting description of the course he took in filling the cavity, by the aid of diagrams on the black board, but the description can hardly be made intelligible in print. [This remark will hold good with regard to all the subsequent speakers on this subject.] Dr. B. said that within the last few months he had changed his whole mode of operating, except in his manner of preparing the teeth before filling. He had been led to do so by a series of experiments in the use of a new foil, which superseded any he had previously used. In his former method of operating, there was always a feverish uncertainty; but now, with the gold he used, and his instruments, which were sharp points, he thought he knew that every point was filled hard enough, so that if any accident happened to the tooth, and any part of the filling was lost, the whole was lost; it did not come out in particles at all. In operating upon front teeth, which had given him more satisfaction than anything else, he restored the shape of every tooth as he went along. The gold he used being chemically pure, was, at his own suggestion, prepared by A. J. Watts & Co., at Utica, and was identical with the "crystal gold foil," prepared by them now, which he obtained fresh, and was not, therefore, subject to the same embarrassment under which others labored who obtained their gold from a distance. He took a sheet of No. 5 of this gold, and, with his hands very dry, rolled it into what might be called a rope, and then cut it into pieces as nearly square as he could. Then he took another sheet, which he cut through the centre, and made it into folds, and so with a third, the folds being made into lengths according to the size of the cavity, longitudinally, and quite small. It had taken him as much labor and care to learn how to use the welding-gold successfully, as it did to learn how to use gold at all in the first place. The instruments he used were very simple, and much reduced in size—three or four summing up the whole in his ordinary operations. The smallest was perhaps the size of a medium-sized pin, the end cut into three delicate points. The largest was about the size of a common knitting-needle, and in that he made four points, not too deep. Then he had another instrument about half way between these two. With the middling-sized instrument he commenced setting up the pellets of gold on the sides, until be got to the very center of the tooth, and until he felt sure that he had introduced enough to fill the tooth from side to side. He did not give much pressure at the commencement; but when the tooth was filled, he pressed the gold, partly sideways and partly by moving the hand back and forth, until it grew harder and harder. It was sometimes said that cohesive gold hardens too quick, but it was not so with him; this he avoided, either by not attempting to harden it when he put in the pieces, or by pressing it lightly and carefully until he had compressed it solid; then, with a finer instrument, shaped so that he could pass it between the walls of the cavity and the portion of the filling he had placed there, he endeavored to free it slightly from the wall. If he found he could do this, he took a smaller pellet, and placed it in this little artificial cavity, and pressed it slowly along until he found he had got nearly to the bottom of the cavity, and then he gave it a hearty push until it was filled up hard. Then he proceeded to the other side, and by the time he had done that, this portion of the cavity was filled, to all intents and purposes, and lay without any motion at all; and thus he was relieved from all uncertainty with regard to the success of the operation. Then he commenced with the other pellets, and filled up from side to side, until he had perhaps two-thirds of the whole length of his cavity, when he took an instrument a little straighter, with a depression in the edge, to hold the pellet, which he inserted at that point, where, from the shape of the instrument, he was able to weld it perfectly, and then he went and filled up his cavity flush with the common surface of the tooth. Then he commenced carefully to restore the shape of the tooth, and finished it up by polishing in any way he pleased. He had never lost a filling inserted in this way, and he was thus relieved from the uncertainty with which he was troubled in years gone by. His patients often said to him—"My dear sir, my teeth look better, you have restored their shape; I am delighted." He supposed they were, and he was himself, so they enjoyed it together. He thought the difficuly that was found in using the cohesive, welding gold, was owing to the fact that it was used in too large quantities, with too large instruments, and the pressure applied too soon. He regarded this gold as very valuable to the profession, as it would enable them to operate in many cases which could not be reached by using unadhesive gold.

In conclusion, Dr. B. said he had never expected to see such a body of men as had met there, gathered together to advance the science of dentistry; he could not express his feeling. There was a time when he had not felt proud of his profession; but now he did. (Applause.) He expected to meet with them as long as he had strength to do so.

Dr. Hawes, of New York, inquired how Dr. Blakesley kept the tooth he was filling dry.

Dr. Blakesley replied that he had accomplished this by using napkins of all sizes, and bibulous paper, which he inserted on the point of his instrument, changing them often.

Dr. E. J. Dunning, of New York, was then called upon and came forward. He said he was not accustomed to describe his operations, but he should be happy to give the Convention any information in his power with regard to his method of operating, if he had anything that was peculiar to himself, which he did not know that he had. He would take, as an illustration, a case, which he regarded as the most difficult they had to meet, and one in which there was the most failures, namely, the approximal cavities of a bicuspid tooth. They presented great difficulties owing to the breadth of the surfaces.

Dr. Perkins, of Milwauke, suggested that Dr. Dunning should take for his example, the second lower molar, posterior surface, to which Dr. D. assented. He said the main difference in the treatment of this case, would be the various methods that would be used to prevent the overflow of the saliva of the lower jaw. And here he would venture one general remark, which seemed very appropriate at this point, and that was, that skill in performing these operations consists more in the preparations that make the work easy, than in the work itself. It would be impossible to fill the cavities of the posterior surfaces of molar teeth, unless the operator could see pretty well; to fill them by the reverse action of mirrors, would be difficult, at least he did not undertake it. In the case referred to he should consider it necessary to carve the surface of the tooth, if it was a case where he thought it best to cut the surface at all, with such an inclination that he could get the light upon it. Forming the cavity was a simple matter of using instruments turned at different angles—simple variations of the angle, or the inside of the cutting instruments. He used the hoe-cutting instrument of different lengths and angles. There were cases were it was necessary to leave one wall of the cavity without an edge; but he had always been careful, and nervously so, about making sure every side of the cavity, if possible. He considered one of the greatest advantages of crystalline gold to consist in this; that they might leave some of the surfaces without cutting them so thin as would be necessary to make them retain foil, leaving a weak edge instead of a strong one. In cases where a free flow of saliva was anticipated, he liked to have a pretty large mass of absorbling matter, napkins or paper, so placed as to cover the sublingual ducts and the buccal ducts, damming them up firmly, so as to prevent the oozing out of moisture or blood, or the mucous secretion from the surface between the teeth. In some cases he used a ligature ; but more frequently, if circumstances were favorable, a wedge of pine wood, so cut as to be retained tightly on the gum, and so that the shape of the teeth might retain it at the edge of the cavity, braced firmly upon the edge of the teeth. It produced considerable pain, but the end obtained, that of securing perfect dryness of the lower part of the cavity, was of great value. The napkin, the paper, the wedge and the ligature, so far as he recollected, comprised all the means he had employed to obviate that difficulty, and he found very few cases where they were not sufficient. He did not like adhesive gold, he wanted a gold that would go into a cavity like kid, or thin muslin, and present as little resistance to the pressure of the instrument as possible, in any direction. After having applied any pressure to the gold, it was a very great object to avoid moving it afterwards, and he had been in the habit, for a year or two, of using a second instrument in his left hand, for the purpose of holding the first few pieces of gold in their place. Even in the case of operating on the lower jaw, he often got his patients to hold the sublingual compress, and used both his instruments. He found a very great advantage in the use of two instruments, in regard to the facility of introducing the gold, for he could make many passes with his left hand that he would otherwise be obliged to make with his right, and in quicker time get the pellet to its place.

A gentleman here inquired how the napkins were retained in their place when both his hands were occupied.

Dr. D. replied that the upper napkin required no retention, being placed over the buccal glands, and adhering to the surface of the teeth. There was very little difficulty in retaining the one placed over the sublingual glands; often it would be retained without assistance, if placed skillfully under the tongue; but he generally let his patient place the first finger of his right hand on the napkin, and keep it in place. He often had to change the napkin, which he did without difficulty. Still, he considered that there were cases where it was next to impossibility to fill a tooth dry. He found a great many bounds to his skill.

Dr. Hawes, of New York, asked if the napkin did not conceal the cavity from the light, and how Dr. D. managed in that case.

Dr. D. replied that that difficulty varied in different mouths. There was only one way to answer that question, and that was, "stretch it!" (Laughter.)

Dr. Hawes.—That is the very question. With an instrument in each hand, and a napkin in the mouth, how do you "stretch it?" (Laughter.)

Dr. D. explained how he did it, by distending the mouth by extending his finger, as he carried the pellet to its place on the instrument. He recommended his brethren to adopt the practice of using a second instrument, if they had not already done so. It overcame the difficulty of hidden cavities, and of broad cavities, where they had no sharp angles to retain the gold in its place when it was first put in. He had found it of great advantage, and had succeeded in overcoming difficulties that he could hardly have conquered in any other way.

Dr. Fuller, of Portsmouth, (N. H.) inquired if, when the tooth was partially filled, the filling got wet by accident, did he think it best to remove it?

Dr. D. replied that he had no objection to the cavity being wet. The evil laid in the injury to the cohesion of the gold when it was packed together, and the finishing up afterward. He thought it best to dry the filling.

Dr. Blakesley inquired what objection the speaker had to cohesive gold?

Dr. D. said he could not use it. He formed his gold into pellets; he did not care about the size of the pellets, only he graduated them to the size of the cavity. He made them globular, like pills. All his pressure was from the centre toward the external walls of the cavity, consolidating in that direction as he went on. His instruments were cubical in their structure, filed down to a plain surface, and turned at different angles. These were plugging instruments without serrated points. The object in serrating them was obtained by sharpening the quadrangles on a stone, which he always did before using gold, to secure the angles of the plane, making it perfect.

Dr. Roberts, of N. Y., gave an account of an operation performed by him on a lateral incisor, which was so much decayed that he could not obtain a wall sufficiently strong to bear the pressure necessary to fill the tooth. He imbedded the tooth, and the one next to it, in gutta percha, up even with the point of the tooth and overhanging on both sides. He then filled it with sponge gold, though he thought he could have done it equally well with adhesive gold, by properly preparing it, and packing it in the same way. He held his thumb upon the gutta percha and filled the tooth, and could use nearly as much pressure as if the tooth had been strong. In such cases it was necessary to fit the gutta percha perfectly, otherwise they would be likely to break the tooth. After filling, he cut out the gutta-percha the shape of the cavity, leaving nothing but the gold for polishing. That was the only way, he thought, in which he could have saved the tooth. He gave credit to Dr. J. S. Clark, of N. O., for giving him the idea.

At the conclusion of Dr. Roberts' remarks, the Convention voted that half an hour of the afternoon session be devoted to a continuance of the discussion on filling teeth, and adjourned to meet at 4 o'clock.

*Afternoon Session.*

The Convention was called to order a few minutes past 4.

Dr. Perine was the first speaker. He said it had long been a desideratum among the profession to secure a composition for filling that should resemble the teeth. They had now a composition—of which he did not claim to be the inventor—which was called the dissolving quartz. After this quartz had been dissolved, he had, in connection with his friend, the inventor, forced it to return to a crystallized state, sufficiently hard to cut his name with it upon glass. The preparation of the quartz was very simple; it was easily dissolved and easily crystallized. The difficulty which he wished to overcome was the contraction of the composition after it was introduced into the teeth. He had experimented upon dead teeth, and by adding a little after the shrinkage, he could make a perfectly hard filling, and give it any shade he desired. He did not wish to present this to the Convention at this time, nor until it was perfected; but, as he was sorry to say, there were those in the dental profession, as in all others, disposed to steal one's thunder, he was anxious that they should give the subject the benefit of their scientific knowledge, and reap what advantage they could from the invention.

Dr. Allport stated that he had received a paper from Dr. Westcott, of Syracuse, which he would hand to the President.

Dr. Allport then proceeded to illustrate his mode of filling. He said that, perhaps, there was no better cavity to take than the one taken by Dr. Dunning, in the morning, with a little alteration. He would take the same cavity, in a small mouth, and a very free flow of saliva, and make a compound cavity of it, embracing not only the approximal but the grinding surface, which was a cavity difficult to fill, and difficult to keep filled after it was filled. Let them imagine the tooth to be the second tooth upon the left side, and the cavity upon the posterior approximal surface. The first thing to be done in filling such a tooth would be to consider the health of the patient; the next, to separate the tooth, and prepare the cavity. In separating the tooth, he would, in the first place, take a chisel, so formed that he could readily cut whatever portion he thought necessary, to save filing, that being rather more unpleasant than cutting the tooth. He endeavored to make the cut much wider on the outside than on the inside in all lower molar teeth, for in this way it was more easily kept clean. The shape should be concave. He then removed the decayed portions in the usual manner. In shaping the cavity, he endeavored to give it the shape of the original tooth as much as possible, but he never sacrificed a good filling to the shape of the tooth. After he had removed the decay, he clipped off the enamel around the edge of the cavity with the chisel, and made it as smooth as possible, slightly counter-sinking it, so that he should have no trouble in making the edge of his filling perfect. He took particular pains to avoid sharp points, which were liable to be broken off in mastication.

The next thing was to know what kind of gold to use. He thought it was necessary, in some places in a tooth, to use gold that was remarkably soft. He had never used any for that purpose which he considered so good as that made by Jones, White & McCurdy, and by Abbey, of Philadelphia. At this point he preferred to use non-adhesive gold. The next thing was to prepare his gold in such a manner as to produce a perfect filling, and consume as little time as possible in manipulating in the mouth. Taking such a portion of non-adhesive foil as might be necessary, perhaps a whole leaf, he folded it into a *block* of suitable size to cover the bottom of the cavity, and extend beyond the edge. He also prepared two similar blocks, large enough to cover the walls of the cavity and extend beyond the edges at the side and top. He then prepared his cylinders in the usual manner, and of two lengths—the one long enough to extend from the anterior wall to beyond the edge, the other of a little more than one-third of the depth of the cavity. For the balance of the operation he prepared a quantity of Watts & Co's crystal gold, or crystal gold foil, which he used in small pieces. In bad cases, where the gums protruded and troubled him with hemorrhage, he used a wedge of some soft wood. If it hurt, that was the business of his patient; it was *his* business to do his duty. In putting in the wedge, it would rise above the cavity, and with a burr, he shaped it to the precise form of the cavity. Then taking his place on the *left* of the patient, with his instruments in convenient position, he put a piece of flax cotton, rolled large in the centre, and small at the ends, under the tongue, where it would lie readily, and also a smaller roll under each cheek, directly over the salivary glands; then placing the chin in the palm of the left hand, he laid a folded napkin, around the back of the tooth, so as to embrace it on three sides, and held it in place by the fore and middle fingers. With a little skill these napkins can be removed and dry ones substituted as often as necessary. In placing the gold in the cavity, he grasped the instrument between the thumb and fore and middle fingers, and pressed the lip back with the little finfier, and first laid the prepared *block* at the bottom; then placed the lateral *blocks* in position, thereby forming a series of layers around the walls, and extending from the bottom to above the top of the cavity; he then filled up *two-thirds* of the space, with the cylinders laid parallel with the floor of the cavity, then packed a sufficient number of cylinders against the anterior wall, and perpendicular to the floor, to hold the lateral blocks firmly in place. The whole inner surface was then thoroughly compressed against the walls of the cavity, and the balance filled up with the crystal gold or adhesive foil, taken up with the point of a serrated instrument, and with it packed into the cavity. After his gold was perfectly packed he filed it off, and the rest of the operation was as usual. In conclusion, Dr. A. exhibited a very simple instrument, the invention of Dr. Garrett, of Wilmington, Delaware, for keeping the napkin over the ducts.

The half hour, to which the debate on this subject was limited, having expired, a motion was made and carried to extend the time.

Dr. Dwinelle remarked that something was said in the morning about riding hobbies. He had large charity for those who rode hobbies, for he rode them himself. Before proceeding to describe any particular mode of filling teeth, he would like to describe a new method which he had adopted for (they must excuse the profanity) damming the ducts. It was a physiological fact, that the muscular effort of throwing the tongue back operated in such a manner upon the sub-lingual ducts, that they would not exude a particle of moisture, it closed them. He took a little piece of gold wire, and in three minutes twisted it in the form of tongs, half an inch long, or shorter. He just clasped the little protruding openings of the ducts with the tongs, let the tongue find its place, and they were effectually closed. The truss principle had before been resorted to by him, and it was a very good one. The old method of bibulous paper and napkins, too, was a very good one.

He would assume that he was required to perform a very difficult operation, which rendered it necessary that the mouth should be kept open an hour or an hour and a half, or even two hours, and he did not want the gums to get wet. He took some strips of fine bird's-eye linen, four inches wide, and tore them into strips from an inch and a half to two and three inches in breadth, and coiled them up into a roll, so that they were about the size of an ordinary sized little finger. He turned them round in a crescent shape, and laid some half dozen of each size of these rolls on his table, in the form of an arch. He wiped the mouth dry and applied a crescent shaped roll to the ducts on the upper side; it would keep its place, adhering to the mucous membrane. He did ditto on the lower side. He then turned to the sublingual ducts and applied a larger roll. In a great many cases the mouths would remain dry during the whole operation; so much so, that oftentimes, in removing the linen, it was found necessary to moisten it, in order to avoid tearing off a portion of the mucous membrane.

Dr. Dwinelle then described an operation which he had performed on an inferior molar tooth, very much decayed, which he took it upon himself to say had been perfectly successful. The lingual wall alone remained, and the gum protruded over into the cavity. [The speaker drew a representation of the tooth on the blackboard.] He wanted to restore the tooth to its original form. In order to do this, he had got to build up the approximal wall artificially. One way would be to drill pits or cells, and fill these pits with crystalline gold, letting them rise above the surface, extending over and interlocking with each other, until he had built up the wall; then he could extend it out and work in the crystalline foil until he had erected the whole superstructure; then he took crystal gold and filled up until he got somewhere near the top of this protuberance, and then filled the whole with adhesive gold, in such a manner that it would correspond with the shape of the tooth, packing it until it crowded the gum back, and gave an entire wall. He then alternated with the crystal gold foil, occasionally pressing this back to give the proper formation to it, until he got up above the gum wall, then he let it double up together, and built directly upon the superstructure. The advantage of this process was that his gold, from beginning to end, was equally consolidated, it was a unit, continually accumulating, from beginning to end—just as one portion of clay added on to another became a part of the whole. It was finished in the usual manner.

Dr. Roberts asked what was to be done if the cavity got wet.

Dr. D. at once imagined himself submerged in the middle of the operation, and proceeded to describe the process of restoration. Every surface, he said, was a receiving surface, except the last one. The first thing, after making the mouth perfectly dry, was to pass a burnisher over the whole of that surface, then press bibulous paper upon it, and dry it anew. He then cut over the surface to get a new surface; then dried it anew. Then he cut the surface backwards and forwards and across, until he had worked it up into a velvet surface. Previous to this, however, he had taken a little strip of crystal gold, No. 1, and annealed it, and got it into particles corresponding with the size of the hole. Having secured his velvet surface, he took a piece of annealed gold, fresh and perfectly adhesive, and laid it on top, and with instruments covered all over with serrations, pressed it down to its place. He then went all over the surface with an instrument terminating in two points, and finally worked the gold into perfect integrity with the other, and was restored to where he was before the flood-gates were opened. He would venture to put those plugs to any test they choose; they would not separate at the point of submerging any sooner than anywhere else.

Dr. D. then went on to describe an operation for restoring lateral incisors that had lost a very large portion of their substance, say one half, which he did by means of diagrams, in a very acceptable manner. His great care was to be sure that his gold, at every stage, was a unit. When the tooth was filled, he said he did not like the gold glittering surface, which reflected light only one way, while he wanted it to reflect light every way. To remedy this, he took a piece of sandal wood, the end moistened and broken up a little, and plunged it into pulverized pumice, and stippled over the entire surface, and then washed it, and so continued until he got a velvet surface with a whitish yellow tint. He had a friend who had four frontal incisors filled as much as the one he had been describing, and he would much prefer the teeth as they were to any artificial teeth.

Dr. Straw, of Bangor, inquired if this filling could be brought to the cutting edge.

Dr. D. replied that it could. In the very case he had been describing the filling occupied a large portion of the front surface. After filling the tooth, he dismissed his patient, but he found afterwards that he articulated only upon the inner surface of the gold. This had bent it round a little, but had not disturbed the joint at all.

Dr. Townsend, of Philadelphia, then described his method of filling cavities in the proximate surfaces of bicuspid teeth, so as to make them stand. It was a difficult thing to prevent the filling in such a tooth from being very imperfect. He said that the way to make difficult things easy was to get at them fairly. He wanted a cavity that he could see into, and there were some cavities that he could not see into unless he had plenty of room to throw light in. In the case supposed, the first thing would be to see if the tooth approximating to the one to be filled was sound, for if he found a carious spot there he knew he would have some chance of getting more room from that tooth for the probe. If he found decay there, he should take care in removing it to preserve the appearance of the tooth on the outer or buccal surface, making the space wider considerably at the sublingual than at the buccal surface. This, for two reasons; one, to preserve the shape of the tooth; the other, because the food is always pushed in towards the tongue, not outwards towards the cheek. The next step was to ascertain how strong the enamel was, and how much he had to cut away. In cleaning out the cavity, one thing was to be guarded against, and that was, not to run to the nerve needlessly. He was satisfied that at this day, when the hobby of killing nerves was ridden so furiously, there were many killed needlessly. His theory was to save the life of the tooth when he could; he, therefore, used instruments with a broad cutting edge, that would not be likely to drop into the nerve cavity. After cutting into the surface, he then endeavored to secure a neat edge at the upper end of the tooth, and then proceeded to cut away the lower edge, having these edges as nearly square as he could, particularly at the margin nearest the edge of the gum. To prevent moisture, he used, besides napkins, in cases of this kind, a wooden wedge, dove-tailed. He used generally orange wood; pine wood he thought too soft, and hickory too unyielding. This should be inserted gently, and by the exercise of proper care, the gums becoming little by little obtunded, it could be placed where it was wanted; this entirely stopped the water above from coming down; and then, having his napkins ready, he was prepared to go to work. The question was to make a filling, and to make a filling at the upper part a little more perfect than anywhere else. He was eclectic in his practice; he used gold in all imaginable shapes. For such cases as this, he took a sheet of gold and folded it lengthwise until he had, as nearly as he could judge, the width of the depth of the cavity, and a little more; he then folded it over upon itself for the length, and then folded it into a block. He folded four or five pieces into the same shape. Then, having the cavity dry, and cutting other gold into little pellets, with sharp points, that he could insinuate anywhere, he proceeded with his second instrument held in the left hand—the dextrous use of which he considered indispensable—to place these blocks of gold in the tooth, pressing them firmly to the upper margin of the cavity, and allowing them to project a little. He packed the gold away with one instrument, while he held it with the other. It was a rule with him that if his first piece became dislodged, he took it away and put in another, and never attempted to use it again, because it would not fit another place. So he went on building up with similar pieces with the lamina pushing outwards. When he got near the end he began to pack in with his pellets, and so on, filling in all the way, until he got down to the end of the cavity. Then he endeavored to insert his instrument between the folds, but usually he found that when he had gone on with the pressure upwards and inwards, there was no place where an instrument could be inserted, except a pointed one, unless a great deal more force was used than was necessary. Some fillings needed to be harder than others. A filling might be perfectly solid, and yet not be as hard as molten gold, and the filling under consideration was one of this character. It might be put in perfectly good, it might have its junction with the walls perfect, and yet not be so solid that the instrument could not be inserted into it. Then he went over the gold with an instrument with four points with which he condensed the whole surface. He had kept the cavity dry, for he considered any condensation after the filling had been wet, as almost, if not entirely useless. He burnished the gold for a considerable time, for he found it had a good effect. He then filed it and burnished again, and so on until he got it down to the edges, and until it was one continuous surface. The burnishing compressed the gold considerably. After he had filed and polished, he went over the surface with a piece of sandal wood, (which he thought as nice wood as could be used,) and very finely pulverized quartz, which was a little finer in its grit than pumice. He then washed perfectly clean and burnished again ; and then took a piece of clean stick and rotten stone, and it was a curious fact that the bone of the teeth was polished more rapidly and smoothly with rotten stone than with pumice, though it was finer, and entirely removed the file marks, so objectionable in an operation of this kind.

On motion of Dr. Straw, of Bangor, the regular order of business was suspended, and Dr. Sanborn, of Andover, was invited to address the Convention on the subject of taking casts.

Dr. Sanborn said, he spoke only because he thought they would find many advantages in knowing the power of the metals with which they took casts. He got the first hint of his method from Dr. Hawes, of New York. He had a sheet iron cup about two and a half inches square, and one and a half deep. He filled this cup with lead, having the plaster cast prepared, and the damper it was, the better; and let it be remembered, that if they could plunge it in the moment they took it from the model, the better it was. The secret was, to begin when the lead was entirely molten, and keep working in from the edges to the centre, so as to have it gradually cool. When it had cooled so that they could scoop it up, and become granulated, he could make no better comparison, than to compare it with hasty pudding when it was done—it would stand up of itself—then take the cast, and plunge it in just right, and the moment the surface of the cast struck the lead, it received a perfect impression, and gave a perfect *fac simile* of the plaster cast. Then if he was in haste, he plunged the cup into water, and took up his tin, and put it into the furnace, melting till it was just molten. While this was going on, they might take a little strip of sheet lead, fold it as they wanted it, and twist it round, so as to fit exactly. Having taken out the plaster cast, he melted the tin, which was to be treated in the same manner with the lead, string it up from the edges to the centre until it granulated, and the moment it began to harden, he poured it in and had the cast. He could take a cast of the mouth in plaster, and be prepared in three-quarters of an hour to strike up as perfect a plate as they could get by any other process

In answer to inquiries, Dr. Sanborn said, he used zinc many years ago, but had found lead far superior, when rightly used; that he regarded pure tin as altogether more advantageous than any combination of metals; and that the impression taken was perfect.

Dr. Spaulding of St. Louis, moved the appointment of a committee, by the chair, to take into consideration the subject of Dental Nomenclature, and report at the next meeting. He said it was evident from the discussions there, that they had no fixed nomenclature, especially for mechanical operations, and he thought the dental profession was far enough advanced now to have a fixed meaning for the terms used in its practice.

Dr. Seabury, of Providence, thought this want had been well met in Prof. Harris' Dental Dictionary.

Dr. Spaulding replied, that notwithstanding the publication of the dictionary, there was still much confusion in terms, and he thought the appointment of such a committee, would call the attention of the profession to the matter, and have a good effect. However, if there was any objection he would withdraw his motion.

The Convention then took up the question of the best

